# Approximate kernel reconstruction for time-varying networks

**DOI:** 10.1186/s13040-019-0192-1

**Published:** 2019-02-06

**Authors:** Gregory Ditzler, Nidhal Bouaynaya, Roman Shterenberg, Hassan M. Fathallah-Shaykh

**Affiliations:** 1 Department of Electrical and Computer Engineering, University of Arizona, Tucson, AZ USA; 2 Department of Electrical and Computer Engineering, Rowan University, Glassboro, NJ USA; 3 Department of Mathematics, University of Alabama at Birmingham, Birmingham, AL USA; 4 School of Medicine, University of Alabama at Birmingham, Birmingham, AL USA

**Keywords:** Time-varying network, Compressive sensing, Gene regulatory, Gene regulatory networks

## Abstract

**Background:**

Most existing algorithms for modeling and analyzing molecular networks assume a static or time-invariant network topology. Such view, however, does not render the temporal evolution of the underlying biological process as molecular networks are typically “re-wired” over time in response to cellular development and environmental changes. In our previous work, we formulated the inference of time-varying or dynamic networks as a tracking problem, where the target state is the ensemble of edges in the network. We used the Kalman filter to track the network topology over time. Unfortunately, the output of the Kalman filter does not reflect known properties of molecular networks, such as sparsity.

**Results:**

To address the problem of inferring sparse time-varying networks from a set of under-sampled measurements, we propose the Approximate Kernel RecONstruction (AKRON) Kalman filter. AKRON supersedes the Lasso regularization by starting from the Lasso-Kalman inferred network and judiciously searching the space for a sparser solution. We derive theoretical bounds for the optimality of AKRON. We evaluate our approach against the Lasso-Kalman filter on synthetic data. The results show that not only does AKRON-Kalman provide better reconstruction errors, but it is also better at identifying if edges exist within a network. Furthermore, we perform a real-world benchmark on the lifecycle (embryonic, larval, pupal, and adult stages) of the *Drosophila Melanogaster*.

**Conclusions:**

We show that the networks inferred by the AKRON-Kalman filter are sparse and can detect more known gene-to-gene interactions for the *Drosophila melanogaster* than the Lasso-Kalman filter. Finally, all of the code reported in this contribution will be publicly available.

## Background

Understanding the dynamical behavior of living cells from their complex genomic regulatory networks is a challenge posed in systems biology; yet it is one of critical importance (i.e., morphogenesis). Gene expression data can be used to infer, or reverse-engineer, the underlying genomic network to analyze the interactions between the molecules. Unfortunately, most of the existing work on reverse-engineering genomic regulatory networks estimates one single static network from all available data, which is often collected during different cellular functions or developmental epochs. The idea that molecular networks are remodeled as a function of time and stage is well understood; this conclusion is supported by the developmental networks of sea urchin embryos [1]. Throughout a cellular process, such as cancer progression or anticancer therapy, there may exist multiple underlying “themes” that determine the functionalities of each molecule and their relationships to others, and such themes are dynamic. In signal processing terms, summarizing gene expression data, that comes from different cellular stages, into one network would be similar to characterizing a non-stationary signal by its Fourier spectrum. Biologically, static networks cannot reveal regime-specific or key transient interactions that lead to biological changes.

One of the challenges of inferring a time-varying network is that there are only a few observations available at each time point. This small sample size is amplified by the high dimension of every sample, leading to a small *n* large *p* problem (i.e., more variables than observations). In particular, the system is under-determined. However, exploiting the fact that molecular networks are sparse, one can use compressive sensing to find a solution. Compressive sensing is concerned with the optimal reconstruction of a sparse signal from an under-determined linear system [2, 3]. Under-determined systems are quite common in computational biology/ecology, and the application of compressive sensing to solve these under-determined systems in nature has been a popular solution [4–6]. Compressive sensing theory states that, under the restricted isometry property (RIP), the optimal sparsest solution of a linear system is equivalent to the minimum *l*_1_-norm solution [2, 3]. Unfortunately, it is almost impossible to check whether a linear system satisfies the RIP condition. In general, the minimum *l*_1_-norm solution can be far from the optimal sparse solution.

In our previous work [4], we addressed the problem of under-sampled sparse systems by proposing a new energy-weighted likelihood function that ensures the convergence of the likelihood function for under-determined systems with unknown covariance. The approach was coined Small sample MUltivariate Regression with Covariance estimation (SMURC) and was applied to infer the wing-muscle gene regulatory networks of the Drosophila Melanogaster during the four phases of its development [4]. However, the estimated networks at every epoch used only the data in the corresponding epoch. In particular, the larval network ignored all the measurements in the previous embryonic phase, and so was the case for the subsequent stages. Other research efforts have been proposed to address the problem of recovering time-varying gene regulatory networks by using dynamic Bayesian models [7], non-parametric Bayesian regression [8], and random graph models [9].

In this contribution, we introduce a new approach to modeling sparse time-varying networks and their applications to gene regulatory networks that are based on our recent work [10, 11]. We start by projecting the Kalman solution onto an “approximately sparse” space by using *l*_1_-regularization. We further expand upon our previous work by using a Kalman smoother. We then explore the *l*_1_-neighborhood for sparser solutions by leveraging our recent compressive sensing technique known as *Kernel RecONstruction* (KRON) [12]. KRON recovers the optimal sparsest solution whether the RIP condition is satisfied or not. However, KRON’s computational complexity is still exponential in the number of parameters *p*. We, therefore, advance Approximate KRON (AKRON) [11], which builds growing neighborhoods of the *l*_1_ solution that moves towards the optimal sparsest solution and eventually reaches it. The size of the neighborhood is tunable depending on the computational resources available. We derive theoretical bounds of optimality. The AKRON Kalman filter is validated on synthetic and real-world data sets.

### The state-space model and Kalman filter

Following the works in [4, 13], we model the network dynamics using a state space model. The system equation is given by a random walk model, which reflects a lack of prior knowledge of the network topological changes. The observation equation is given by a first-order differential equation, whose parameters reflect the strength and sign of interactions (positive and negative are activating and repressing, respectively) [14]. The state space model of the incoming edges, $\mathbf {a}_{i} \in \mathbb {R}^{p}$, for gene *i* can be shown to be [13]

1 $$\begin{array}{*{20}l} \mathbf{a}_{i}(k+1) =& \mathbf{a}_{i}(k)+\boldsymbol{w}_{i}(k).  \end{array} $$

2 $$\begin{array}{*{20}l} \mathbf{y}_{i}(k) =& \mathbf{X}^{\mathrm{T}}(k)~ \mathbf{a}_{i}(k)+\boldsymbol{v}_{i}(k),  \end{array} $$

where *i*=1,⋯,*p* and *p* is the number of genes. $\boldsymbol {X}(k) \in \mathbb {R}^{p \times n}$ is the gene expression matrix at time *k*. **y**_*i*_(*k*) is the rate of change of expression of gene *i* at time *k*. ***w***_*i*_(*k*) and ***v***_*i*_(*k*) are the process and observation noise, respectively. These noise processes are assumed to be zero mean Gaussian noise processes with the known covariances ***Q***_*k*_ and ***R***_*k*_, respectively, and uncorrelated to the state vector ***a***_*i*_(*k*). The full connectivity matrix, ***A***(*k*), can be recovered by simultaneous parallel recovery of its rows $\boldsymbol {a}_{i}^{t}(k)$ at every time instant *k*. Thus, we can process each gene in parallel. The Kalman filter can be used to track ***a***(*k*) [13, 15]; however, this is only if the system is observable. The problem with using a Kalman filter in our setting is that the system is under-determined (i.e., more variables than equations, *p*>*n*). This problem, however, can be circumvented by taking into account the sparsity of the vector ***a***_*i*_(*k*). Since each gene in the genomic regulatory network is governed by only a small number of other genes, these networks are known to be sparse. Furthermore, we have also experimented with a Kalman Smoother that is applied after the Kalman filter. Note that the Kalman Smoother is optional. A Kalman smoother can reduce the covariance of the optimal estimate. We implemented Rauch et al.’s Kalman smoothing algorithm [16] and we compared AKRON-Kalman both with and without smoothing.

**Fig. 1:**
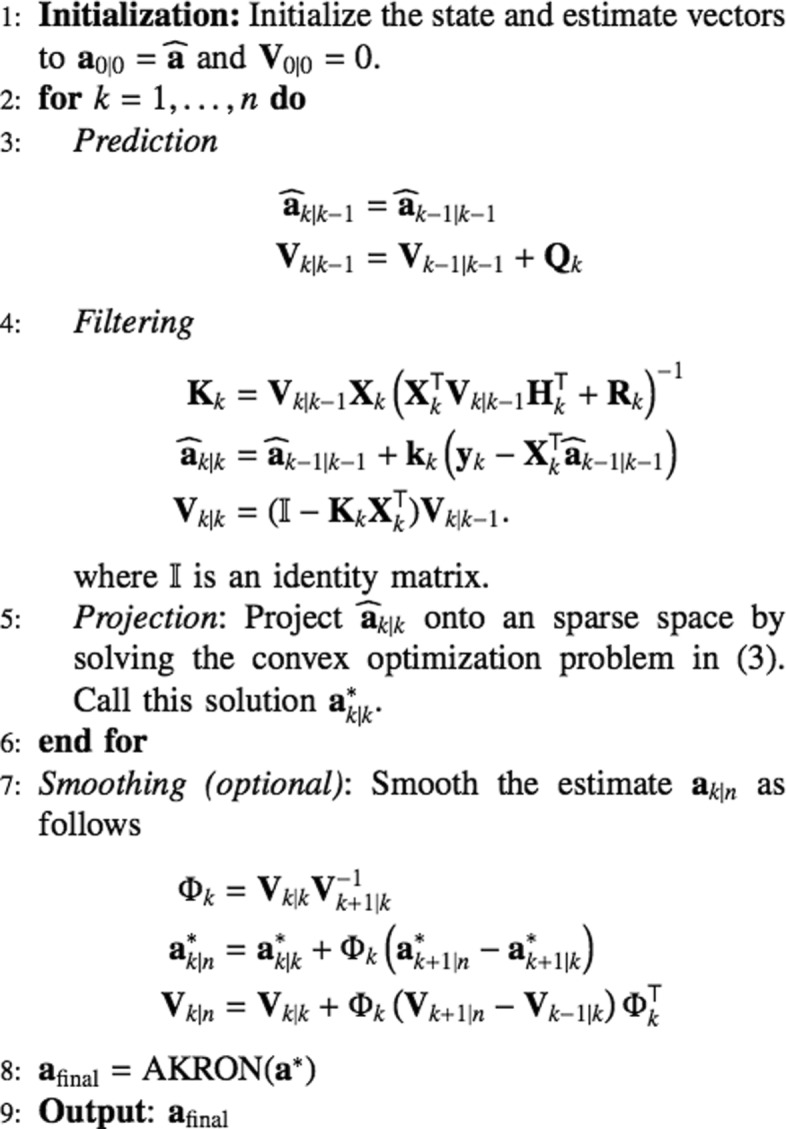
AKRON-Kalman Tracker+Smoother

The pseudo code for the proposed Kalman filtering approach is shown in Fig. 1.

### Constrained Kalman filtering

It is known that the connectivity of the edges in gene regulatory networks [13] is sparse. Unfortunately, the output of the Kalman filter is likely not going to be sparse. We first start by projecting the Kalman solution at time *k*, ***a***_*k*|*k*_, onto the set of “approximately sparse” vectors by solving the following Lasso problem [17]:

3 $$ \boldsymbol{a}_{k|k}^{*} = \arg\min_{\boldsymbol{a}\in \mathbb{R}^{p}} \left\{(1 - \alpha) \left\|\boldsymbol{a}_{k|k} - \boldsymbol{a}\right\|_{2}^{2} + \alpha \|\boldsymbol{a}\|_{1}\right\},   $$

where *α*∈[0,1] controls the tradeoff between the Kalman estimate and sparsity. An *α* close to zero will result in a solution that is close to the Kalman estimate, but that may not be sparse. The opposite happens when *α* is close 1, which will produce a sparser solution, but may be far from the Kalman estimate. This is achieved by minimizing the reconstruction error (i.e., the first term in (3)).

### AKRON: a search for a sparser solution

Consider the following *l*_0_-optimization problem, which finds the optimal sparsest solution in a linear under-determined system.

4 $$\begin{array}{*{20}l} \boldsymbol{x}^{*} = &\arg \min_{\boldsymbol{x} \in \mathbb{R}^{p}} \|\boldsymbol{x}\|_{0} \\ & \text{s.t.} \; \boldsymbol{\Phi}\boldsymbol{x} = \boldsymbol{y}  \end{array} $$

where ∥·∥_0_ is the *l*_0_-norm, which is defined as the support of the vector, $\boldsymbol {x} \in \mathbb {R}^{p}$, $\boldsymbol {y} \in \mathbb {R}^{n}$, and $\boldsymbol {\Phi } \in \mathbb {R}^{n \times p}$. We consider the scenario where *p*≫*n* and denote in the sequel *s*=*p*−*n*. Without loss of generality, ***Φ*** is assumed to be full-rank. Compressive sensing theory [3] shows that, under the Restricted Isometry Property (RIP) condition on the matrix ***Φ***, the *l*_1_-norm solution is equivalent to the *l*_0_-norm solution. Unfortunately, it is impossible to check if the RIP condition is satisfied for a given matrix. Despite this strict condition, *l*_1_ has been routinely used to find a sparse solution in systems of the form (4).

The proposed Approximate Kernel RecONstruction (AKRON) is an approximation to computationally complex Kernel RecONstruction (KRON) problems [12]. KRON is able to achieve an exact solution to (4), but the algorithm becomes computationally expensive for typically *p*>15. AKRON, detailed below, is introduced to balance the trade-off between the computational resources that are available and the accuracy of the reconstruction.

The Kalman filter estimate is first sparsified by incorporating *l*_1_ regularization in (3) (line 5 of Fig. 1). However, the *l*_1_ projection is not guaranteed to be the optimal sparsest solution. AKRON-Kalman filter (AKRON-KF) starts off from the *l*_1_-regularized Kalman estimate in (3). Then, the *s*=*p*−*n* smallest elements of the *l*_1_ projection in (3) are set to zero. The logic behind this strategy is to use the *l*_1_-projection to guess the position of the zeros in the optimal solution. Given that the kernel of the system matrix ***Φ*** in (3) has dimension *s*, we know that if *s* zero locations are correctly set, then the optimal sparsest solution can be exactly found by solving the linear system in (4) [12].

Following this reasoning, AKRON finds a sparser solution by exploring *δ*-neighborhoods of the *l*_1_-projection. The central idea behind AKRON’s *δ*-neighborhoods is as follows: (i) find the indices with the (*s*+*δ*) smallest magnitudes of the *l*_1_ solution, (ii) set exactly *s* of these indices to zero, (iii) re-solve the system ***Φx***=***y***. All the possible $s+\delta \choose s$ combinations of the smallest elements in the solution of (3) are evaluated. This idea can also be viewed as a “perturbation” of the *l*_1_ approximation to make it closer to the *l*_0_-norm. The size of the neighborhood *δ* is tunable depending on the computational power available, and vary from 0 (*l*_1_-approximation) to *n* (KRON, i.e., perfect reconstruction).

**Example**: To understand the AKRON algorithm and illustrate the importance of the *δ*-neighborhoods, we present a simple numerical example. Consider the following randomly generated noiseless system as in (4):

$$\begin{array}{*{20}l} \boldsymbol{\Phi} =& \left(\begin{array}{ccccc} -0.4588 & 1.5977 & -0.8724 & -0.1121 & -1.3068 \\ 0.2942 & 3.0954 & -1.0530 & 0.3454 & 1.5257 \\ -0.1948 & -0.7558 & -0.9756 & 0.1549 & 0.9586 \\ \end{array} \right); \\ \boldsymbol{y} =& \left(\begin{array}{ccc} -1.2316 & 1.1739 & 0.8135 \\ \end{array} \right)^{T} \end{array} $$

The optimal *ℓ*_0_-norm sparsest solution is given by

5 $$  \boldsymbol{x}^{*} = \left(\begin{array}{ccccc} 0 & 0 & 0 & -1.2372 & 1.04858 \\ \end{array} \right)^{T}  $$

The *ℓ*_1_ solution, which solves (4), is given by

$$ \widehat{\boldsymbol{x}}_{1} = \left(\begin{array}{ccccc} 0.0 & -0.034 & 0.047 & 0.0 & 0.870 \\ \end{array} \right)^{T}  $$

Clearly, the *l*_1_-solution is not as sparse as the optimal solution and has incorrect zero locations. We have *n*=3,*p*=5 and thus *s*=2. If we choose *δ*=1 the AKRON considers the *s*+*δ*=3-smallest magnitudes of $\widehat {\boldsymbol {x}}_{1}$, which are located at indices 1, 2 and 4. We set *s*=2 locations to zero among these 3 indices. We consider all ${s+\delta \choose s} = {3 \choose 2} = 3$ combinations of two zeros in indices 1, 2 and 4 of $\widehat {\boldsymbol {x}}_{1}$. The combination of indices 1 and 2 set to zero leads to the sparsest optimal solution ***x***^∗^ in (5). Thus, in this case, the *ℓ*_1_-norm solution is sub-optimal; but by considering a *δ*=1-neighborhood of this approximation, AKRON is able to exactly recover the sparsest optimal *l*_0_-solution.

In the noisy case, where the constraint in (4) is replaced by ∥***Φx***−***y***∥≤*ε*, with *ε* being a given noise threshold level, the neighborhood *δ* is chosen adaptively as follows: set the *s* smallest magnitudes of the *l*_1_ solution to zero; compute the observation error ∥***Φx***−***y***∥. If this error is smaller than the energy of the noise, we adopt this solution. Otherwise, the next smallest element is set to zero and the error is recalculated.

In the following propositions, we investigate under which assumptions on the entries of the *l*_1_ solution and its closeness to the *l*_0_ solution, will AKRON yield the optimal *l*_0_ solution.

#### **Proposition 1**

Consider the system in (4) with the optimal *l*_0_-solution ***x***^∗^ having *k*>*s* zeros. Consider the *l*_1_-solution, ***x***_1_, and assume that ∥***x***_1_−***x***^∗^∥_2_≤*ε*. Let *J* denote the number of indices *j* such that

6 $$\begin{array}{*{20}l} |(\boldsymbol{x}_{1})_{j}| \leq \frac{\epsilon}{\sqrt{k-s+1}}.  \end{array} $$

Then, by choosing *δ*≤*J*−*s*, AKRON yields the optimal *l*_0_-solution.

#### *Proof*

Let *Θ* and $\overline {\Theta }$ be the index sets of zero and non-zero entries in the *l*_0_-solution ***x***^∗^, respectively. We have |*Θ*|=*k*. We need to show that at least *s* indices in *Θ* are where (6) holds. To prove this fact, assume the opposite, i.e., at least (*k*−*s*+1) indices in *Θ* are such that

$$|(\boldsymbol{x}_{1})_{j}| > \frac{\epsilon}{\sqrt{k-s+1}}. $$

However, in this case, we have

$$\|\boldsymbol{x}_{1} - \boldsymbol{x}^{*}\|_{2} > \sqrt{k-s+1}\frac{\epsilon}{\sqrt{k-s+1}} = \epsilon, $$

which contradicts the assumption ∥***x***_1_−***x***^∗^∥_2_≤*ε*. □

The following proposition derives an upper bound for $\delta \in \mathbb {N}_{+}$ when the non-zero elements of the optimal *l*_0_-solution are bounded from below. We first need the following Lemma.

#### **Lemma 1**

Consider the system in (4) with the optimal *l*_0_-solution ***x***^∗^ and approximate *l*_1_-solution ***x***_1_. Denote by *Θ* and $\overline {\Theta }$ the index sets of zero and non-zero entries in ***x***^∗^, respectively. Assume that ∥***x***_1_−***x***^∗^∥_2_≤*ε*. Let *R* be the number of indices $j \in \overline {\Theta }$ such that |(x_1_)_*j*_|≤*ε*. If *δ*=*R*, then AKRON yields the optimal solution.

#### *Proof*

To obtain the sparsest *l*_0_-solution, it is sufficient to choose *s* zeros in “correct places”, i.e., with indices in *Θ*. Recall that AKRON sets *s* out of the smallest-magnitude (*s*+*δ*) entries in ***x***_1_ to zero. Therefore, AKRON will yield the optimal solution if out of these (*s*+*δ*) smallest-magnitude entries, there are at least *s* entries from *Θ*. But all entries from *Θ* have |(***x***_1_)_*j*_|≤*ε*, for otherwise the assumption ∥***x***_1_−***x***^∗^∥_2_≤*ε* would be violated. This means that only those entries from $\overline {\Theta }$, which also satisfy |(***x***_1_)_*j*_|≤*ε*, could be chosen by AKRON, and there are only *R* of them. Hence *δ*=*R* will yield the optimal *l*_0_-solution.

Lemma 1 provides a sufficient condition on *δ* for optimality of AKRON, namely, if *δ*=*R*, then we are guaranteed the optimal *l*_0_-solution. Since this condition is not necessary, we could reach optimality with *δ*≤*R*. □

#### **Proposition 2**

Consider the system in (4) with the optimal *l*_0_-solution ***x***^∗^ and approximate *l*_1_-solution ***x***_1_. Let $\overline {\Theta }$ be the set of indices of non-zero elements in ***x***^∗^. Assume that the non-zero entries of x^∗^ are bounded from below, i.e.,

$$\left|\left(\boldsymbol{x}^{*}\right)_{j}\right| \geq \eta,~ \text{for all}~ j \in \overline{\Theta}~ \text{and for some}~ \eta > 0. $$

Assume further that ∥***x***_1_−***x***^∗^∥_2_≤*ε*. If *ε*<*η*, then $\delta \leq \left (\frac {\epsilon }{\eta - \epsilon }\right)^{2}$ yields the optimal *l*_0_-solution. In particular, if $\epsilon < \frac {\eta }{2}$ then *δ*=0 suffices.

#### *Proof*

Since |(***x***^∗^)_*j*_|≥*η* for $j \in \overline {\Theta }$, we have

$$\left\|\boldsymbol{x}_{1}-\boldsymbol{x}^{*} \right\|_{2} \geq (\eta-\epsilon)\sqrt{R}\geq(\eta-\epsilon)\sqrt{\delta}, $$

where we used the fact that *δ*≤*R* from Lemma 1. Thus, $\epsilon \geq \left \| \boldsymbol {x}_{1}-\boldsymbol {x}^{*} \right \|_{2} \geq (\eta -\epsilon)\sqrt {\delta }$, which completes the proof. □

Although Propositions 1 and 2 derive theoretical bounds for the choice of the neighborhood radius *δ* to recover the optimal sparsest solution, we found in our experiments below that relatively small values of *δ* are sufficient to achieve a balance between desired accuracy and computational complexity.

## Results

In this section, we present an empirical analysis of the AKRON-KF and its smoother, including comparisons to other approaches proposed for detecting the relationships between different genes in a molecular network. The experiments include a number of carefully designed synthetic data sets, as well as a real-world data set, namely the fruit fly.

### Overview of experimental protocols

Our experiments are conducted on real-world and synthetic data. The advantage of the synthetic data are that the ground truth networks are known; therefore, we can calculate different statistics about the reconstruction error of the network. Unfortunately, we do not have a clear view of the “ground truth” for real-world data. Therefore, we use findings from the life sciences that have studied these networks and were able to infer gene-to-gene relationships that are well established [18].

Our experiments make use of the following algorithms for a sparse reconstruction of a time-varying network:

- *l*_1_-KF(S): This algorithm is the output of the Kalman filter with the *l*_1_ projection applied to the state vector. The (S) indicates whether the smoother was implemented.
- AKRON-KF(S): This is the proposed approach using the output of *l*_1_-KF(S) to seed AKRON. It is also implemented with and without the smoother.

Both of the above algorithms can reconstruct a network that represents the interactions between genes. We compute the true positive (*TP*), true negative (*TN*), false positive (*FP*) and false negative (*FN*) rates. These rates are summarized through accuracy (acc), sensitivity (sen), specificity (spe), and Matthew’s correlation coefficient (mcc), which are defined below.

$$\begin{array}{*{20}l} \text{acc} &= \frac{TP + TN}{TP+TN+FP+FN}, \\ \text{sen} &= \frac{TP}{TP+FN}, \\ \text{spe} &= \frac{TN}{TN+FP}, \\ \text{mcc} &= \frac{TP \cdot TN - FP \cdot FN}{\sqrt{(TP+FP)\cdot(TP+FN)\cdot(TN+FP)\cdot(TN+FN)}}. \end{array} $$

Matthew’s correlation coefficient provides a more balanced statistic for examining the overall trade-offs between the different rates (i.e., *TP*, *TN*, *FP*, and *FN*).

### Results on synthetic data

Synthetic time-varying networks are simulated to evaluate the efficacy of the proposed AKRON-KF(S) on data that we have complete control over. All results in this section are presented as the average over 25 monte carlo simulations. Averaging is performed because there could be a large degree of variation in the time-varying networks that are randomly generated.

**Fig. 2:**
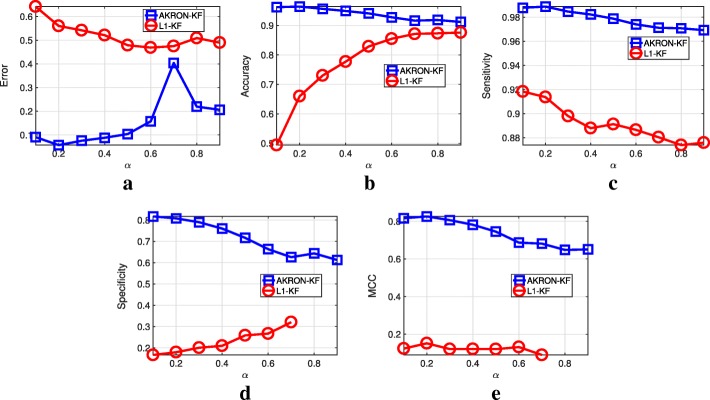
Comparison of AKRON-KF and *l*_1_-KF on a synthetic data with 25 genes over four time points and with 85% sparsity. All results are averaged over 25 monte carlo simulations. **a** Error. **b** Accuracy. **c** Sensitivity. **d** Specificity. **e** MCC

First, we evaluate the impact of *α* in (3) on the estimation of ***a***_*k*|*k*_ and the reconstruction errors of *l*_1_-KF and AKRON-KF. The experiment is configured as follows: a 25-gene network evolves over four-time points by simulating a random walk; all networks being 85% sparse. At each time step, there are nine observations that are available. Figure 2 shows the effect of *α* on the reconstruction errors of AKRON-KF and *l*_1_-KF. Clearly, AKRON-KF is the better performer across the statistics that we collected. Thus, AKRON significantly improves the previous implementation of sparse Kalman filters for time-varying networks. Furthermore, AKRON-KF detects the location of the edges in the simulated networks (see Fig. 2b). Simply using the solution from *l*_1_-KF for a small *α* is not enough to find the location of the zeros. In fact, *α* needs to be close to one to achieve a high accuracy at edge detection (i.e., (3) will place a large weight on the *l*_1_ penalty and a small weigh on the error). Given these results, we choose *α*=0.2 for the remainder of the experiments since this value provides a reasonable trade-off between the different statistics that were assessed. Figure 2e shows that AKRON-KF is also superior to Lasso-KF as assessed by Matthew’s correlation coefficient, which provides a balanced measure.

Second, we expand the synthetic experiments to evaluate the impact of *δ* in AKRON and the dimensionality of the kernel of ***X***, and we also evaluate the impact that the smoothing has on the reconstruction of the networks. We simulated three different kernel dimensions and values for *δ*. Table 1 shows the outcome of these experiments. The entries in the table are presented as *A*/*B*, where *A* and *B* are the results from AKRON-KF and *l*_1_-KF, respectively. The table is also divided in half to separate the results for the Kalman filter and Kalman smoother. Similar to the first experiment, we observe that AKRON-KF typically outperforms *l*_1_-KF in nearly every statistic and the results can be quite significant. Furthermore, systems with a large kernel dimension (i.e., large *p* small *n*) benefit significantly from a larger value of *δ*. For example, consider a network that is 9×50×4. *δ*=1 provides a little reduction in error; however, increasing *δ* to 3, significantly reduces the error and increases the other statistics. Finally, we observe that smoothing improves the error of the system; however, we should note that this improvement comes at a computational cost.

**Table 1:** Results on simulated networks

		AKRON-KF			AKRON-KFS		
		*δ*=1	*δ*=2	*δ*=3	*δ*=1	*δ*=2	*δ*=3
err	9×11×4	2.27/131.42	1.83/119.78	2.74/128.54	0.68/133.65	1.38/122.99	1.21/128.05
	9×25×4	7.86/62.42	25.49/58.88	16.84/63.87	1.72/64.72	8.99/69.45	4.85/68
	9×50×4	17.06/18.06	10.92/18.25	4.94/18.81	7.11/22.02	12.59/21.35	2.74/22.47
acc	9×11×4	97.52/57.6	98.57/54.87	98.59/56.06	98.23/58.28	98.53/57.98	99.33/57.04
	9×25×4	96.31/63.25	97.49/65.17	98.17/65.43	97.82/77.12	98.84/77.23	99.46/78.54
	9×50×4	97.79/85.74	98.87/85.35	99.22/85.05	98.31/93.99	99.05/93.99	99.52/93.9
sen	9×11×4	98.52/67.25	98.92/65.69	98.62/66.22	99.1/65.89	98.68/65.29	99.34/64.98
	9×25×4	98.69/89.51	98.7/90.61	98.95/91.67	99.28/89.48	99.37/89.41	99.65/90.18
	9×50×4	99.32/98.73	99.59/98.78	99.67/98.72	99.48/98.27	99.63/98.24	99.78/98.23
spe	9×11×4	96/45.58	97.96/41.55	98.55/43.94	96.83/43.19	98.29/42.53	99.3/42.17
	9×25×4	82.55/17.32	89.28/17.79	92.33/19.42	88.46/20.71	94.97/20.01	98.05/22.18
	9×50×4	48.16/7.17	68.95/6.95	78.78/6.31	57.77/9.37	73.93/8.17	87.62/7.72
mcc	9×11×4	94.6/6.1	95.23/11.33	97.17/10.56	96.7/12.95	97.46/8.63	97.52/8.61
	9×25×4	84.74/12.08	89.1/13.45	92.88/11.88	90.76/7.92	97.76/8.76	95.47/10.86
	9×50×4	54.44/13.33	65.12/13.99	81.71/13.3	58.54/7.05	79.02/9.81	86.51/8.11

Each entry in the table reports two numbers separated by “/”. The number on the left is the result of AKRON and the number on the right is the result of Lasso-KF. The simulation is performed with a 15% network density. The dimensions *A*×*B*×*C* can be interpreted as *A* samples with *B* genes across *C* timestamps

### Results on Flybase

The application of interest is the inference of the time-varying wing-muscle genomic network of the Drosophila Melanogaster (fruit fly). The Drosophila’s microarray dataset originally consists of 4028 genes taken over 66 different time points [18]. The data includes 4 stages of the Drosophila’s life: embryonic (samples 1 through 30), larval (samples 31 through 40), pupal (samples 41 through 58), and adulthood (samples 59 through 66). Flybase hosts a list of undirected gene interactions [19]. We set *α*=0.2 based on the experiments in the previous section for *l*_1_-KF and AKRON-KF.

In this application, we considered a list of 11 genes that are responsible for the wing muscle development, which has been considered by many researchers before [7–9, 20]. The embryonic, pupal, and larval stages are undersampled to 9 observations in each stage that were used in the reconstruction of the 11-gene network in each developmental epoch. All 8 time points were used in the adulthood period. To summarize, the reconstruction of the connectivity matrix uses 9 samples in the embryonic, pupal, and larval developmental stages and 8 samples in the adulthood developmental stage. The 11 gene network was reconstructed throughout each of the four developmental stages using AKRON-KF and AKRON-KFS.

**Fig. 3:**
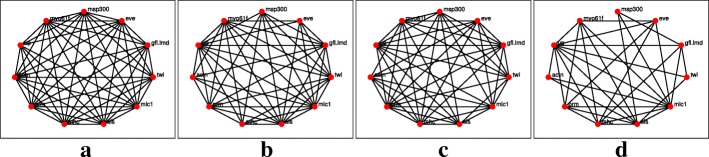
Reconstructed networks for the *l*_1_-KF across the four time stages. Edges in the network represent either the suppression of a gene or excitation of a gene, or one gene excites while the other suppresses. **a** Embryonic. **b** Larval. **c** Pupal. **d** Adult

**Fig. 4:**
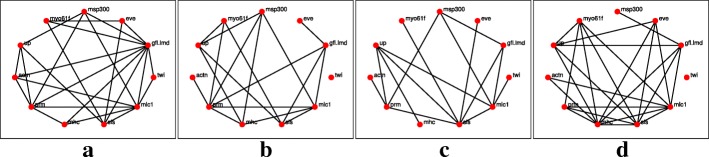
Reconstructed networks for the AKRON-KF across the four time stages. Edges in the network represent either the suppression of a gene or excitation of a gene, or one gene excites while the other suppresses. **a** Embryonic. **b** Larval. **c** Pupal. **d** Adult

The networks reconstruction using the *l*_1_-KF and AKRON-KF are shown in Figs. 3a-d and 4a-d, respectively. For clarity, the displayed networks do not show the strength of the interaction, only that there is an interaction detected by one of the algorithms. The AKRON-KF tracker results in clearly sparser networks than the *l*_1_-KF. AKRON-KF was able to find all the connections reported in Flybase: (Actn,prm) appears in the embryonic, larval, and pupal stages, (Actn,up) appears in all four stages, (up,mhc) appears in the embryonic, larval, and pupal stages, (up,sls) appears in the embryonic, larval, and pupal stages and (sls,mhc) appears in the embryonic and larval stages. The other two connections appear through one medium gene (Actn,sls) appears in the embryonic, pupal and adulthood phases through one additional gene and (twin, eve) appears through one or more additional genes only in the embryonic and larval phases.

**Fig. 5:**
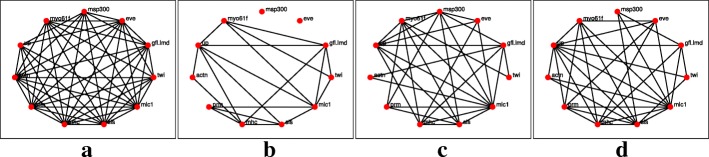
Reconstructed networks for the *l*_1_-KF with a smoother across the four time stages. Edges in the network represent either the suppression of a gene or excitation of a gene, or one gene excites while the other suppresses. **a** Embryonic. **b** Larval. **c** Pupal. **d** Adult

**Fig. 6:**
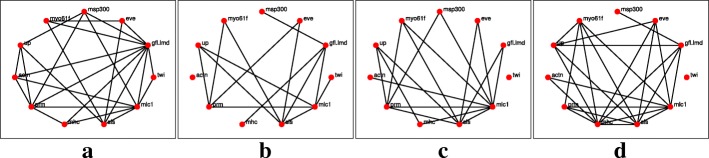
Reconstructed networks for the AKRON-KF with a smoother across the four time stages. Edges in the network represent either the suppression of a gene or excitation of a gene, or one gene excites while the other suppresses. **a** Embryonic. **b** Larval. **c** Pupal. **d** Adult

**Fig. 7:**
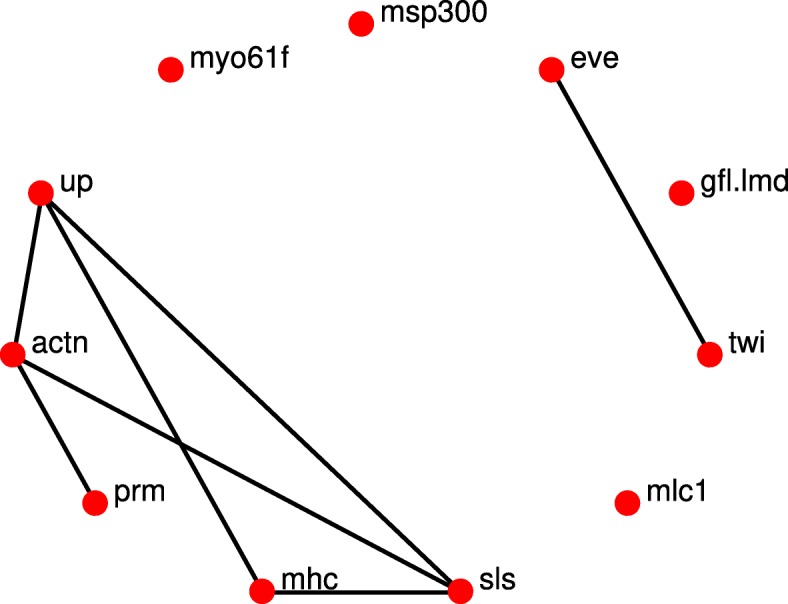
The known undirected gene interactions in the Drosophila’s 11-gene wing muscle network as determined from Flybase [18]

The networks reconstruction using the *l*_1_-KF and AKRON-KF with smoothers are shown in Figs. 5a-d and 6a-d, respectively. The smoothing provides very similar network topologies to the ones without the smoother; however, we did observe that the networks were sparser in the larval stage (see Figs. 4b and 6b). Note that while Fig. 7 is considered the ground truth, the could exist relationships that have not yet been discovered. These statistics are shown in Table 2, which again shows the benefit of using the AKRON-KF over the *l*_1_-KF.

**Table 2:** Results of the AKRON-KF and *l*_1_-KF on the real-world Drosophila Melanogaster data set

	Algorithm	acc	sens	spec	mcc
AKRON	*δ*=1	35.54	93.94	13.64	10.55
	*δ*=2	36.16	94.07	13.75	10.97
	*δ*=3	38.02	95.71	14.53	14.53
	*ε*=0.0005	49.59	90.35	13.28	5.67
	*L*_1_-KF	38.43	88.69	11.71	0.59

Table 3 lists all previous algorithms that were applied to this genetic network. Only the AKRON-KF, *l*_1_-KF [13], SMURC [4] and Dynamic Bayesian networks [7] considered time-varying networks; and, hence, were able to distinguish the different phases in the network. The other algorithms (minimum description length [20], random graph model [9], and nonparametric Bayesian regression [8]) assumed a stationary network, and hence it is not clear at which stage the detected connections develop. The AKRON-KF along with the *l*_1_-KF are the only algorithms able to recover all known interactions and specify the developmental stage where these interactions occur. Although the *l*_1_-KF also finds all reported interactions, the networks are denser (less sparse) than the AKRON-KF. With regard to the MDL results in Table 2, we have not reported the interations. The MDL authors in [20] inferred a single network, using all 66 time points, that characterizes the entire Drosophila’s life cycle. In particular, the MDL approach is stationary and does not differentiate between the phases or time-varying epochs of the data. We wanted to report the MDL findings as they are in the literature. Notice that AKRON used less points and found more correct interactions.

**Table 3:** Detection of the known gene interactions in Flybase (E: embryonic, L: larval, P: pupal and A: adulthood)

	(*prm,Actn*)	(*sls,mhc*)	(*mhc,up*)	(*sls,Actn*)	(*sls,up*)	(*twi,eve*)	(*up,Actn*)
AKRON-KFS	$\checkmark $ (E,L,A)	$\checkmark $ (A)	$\checkmark $ (L,P,A)	$\checkmark $ (E,P,A)	$\checkmark $ (E,L,P,A)	$\checkmark $ (E,L)	$\checkmark $ (E)
AKRON-KF	$\checkmark $ (E,L,A)	$\checkmark $ (A)	$\checkmark $ (L,P,A)	$\checkmark $ (E,P,A)	$\checkmark $ (E,L,P,A)	$\checkmark $ (E,L)	$\checkmark $ (E)
LASSO-Kalman [13]	$\checkmark $ (E,L,P)	$\checkmark $ (E,L)	$\checkmark $ (E,L,P)	$\checkmark $ (E,L,P)	$\checkmark $ (E,L,P)	$\checkmark $ (E,L,P,A)	$\checkmark $ (E,L,P,A)
SMURC [4]	$\checkmark $ (A)	$\checkmark $ (A)	$\checkmark $ (L)	$\checkmark $ (L)	$\checkmark $ (E)	$\checkmark $ (P)	×
MDL [20]	$\checkmark $	$\checkmark $	×	×	×	$\checkmark $	×
Random graph model [9]	×	×	$\checkmark $ (E,L,P,A)	$\checkmark $ (P,A)	$\checkmark $ (E,L,P,A)	×	×
Dyn. Bayes. netw. [7]	×	$\checkmark $ (E,L,P,A)	×	×	×	×	×
Nonpar. Bayes. [8]	×	×	×	×	×	$\checkmark $ (E)	×

## Conclusion

In this work, we addressed the problem of inferring time-varying molecular networks as a tracking problem that can be solved using the Kalman filter. The major difficulty, however, is that there is not a sufficient number of observations at each time point, which makes the state-space model unobservable and the tracking senseless. Fortunately, molecular networks are known to be sparse because the dynamics of every gene are governed by only a small number of genes. By incorporating the sparsity condition, we show that the tracking problem becomes feasible.

We presented the AKRON Kalman filter, which builds on our previous work on the Lasso-Kalman filter (*l*_1_-KF). Our proposed approach leverages the AKRON algorithm to find a sparser solution that is more representative of the ground truth. The proposed tracker/smoother first computes the output of *l*_1_-KF; then explores growing neighborhoods of the *l*_1_-projection to look for sparser solutions, eventually reaching the optimal sparsest estimate. The size of these neighborhoods is a tunable parameter that depends on the computational power available. AKRON-KF was benchmarked on synthetic and real-world data against *l*_1_-KF. The results demonstrate that the proposed approach is better at recovering sparse time-varying networks than *l*_1_-KF. Not only was the reconstruction error of the proposed approach lower than *l*_1_-KF, but it was also better at detecting whether an edge exists in a network. AKRON-KF tracker was applied to infer the wing muscle gene regulatory network of the *Drosophila Melanogaster* during four developmental phases of its life cycle, and successfully identified all seven known interactions reported in Flybase. We should also note that our proposed approach will work for time-series networks that have more than four times steps and sparsity levels

Our future work includes applying the AKRON-KF to other types of data, particularly data related to different types of cancers to create a predictive network biomarker for clinical outcome. These ideas have applicability in translational clinical cancer research, basic cancer research, and in network-based drug discovery.

## References

[1] Sethi AJ, Wikramanayake RM, Angerer RC, Range RC, Angerer LM (2012). Crosstalk regulates endomesoderm segregation in sea urchin embryos. Science.

[2] Foucart S, Rauhut H. A mathematical introduction to compressive sensing. Birkhäuser. 2013.

[3] Fornasier M, Rauhut H. Compressive sensing. In: Handbook of Mathematical Methods in Imaging: 2011.

[4] Bayar B, Bouaynaya N, Shterenberg R (2016). SMURC: High-dimension small-sample multivariate regression with covariance estimation. IEEE J Biomed Health Inform.

[5] Koslicki D, Foucart S, Rosen G (2014). WGSQuikr: fast whole-genome shotgun metagenomic classification. PLoS ONE.

[6] Koslicki D, Rosen G, Foucart S (2013). Quikr: a method for rapid reconstruction of bacterial communities via compressive sensing. Bioinformatics.

[7] Robinson JW, Hartemink AJ (2010). Learning non-stationary dynamic bayesian networks. J Mach Learn Res.

[8] Miyashita H, Nakamura T, Y Ida TM, Kaburagi T. Nonparametic Bayes-based Heterogenious Drosophila Melanogaster Gene Regulatory Network Inference: T-process Regression. In: International Conference on Artificial Intelligence and Applications: 2013. p. 51–8.

[9] Go F, Hanneke S, Fu W, Xing EP. Recovering Temporally Rewiring Networks: A Model Based Approach. In: International Conference on Machine Learning: 2007. p. 321–8.

[10] Carluccio V, Bouaynaya N, Ditzler G, Fathallah Shaykh HM. The AKRON-Kalman Filter for Tracking Time-Varying Networks. In: IEEE International Conference on Biomedical and Health Informatics: 2017.

[11] Ditzler G, Bouaynaya N, Shterenberg R (2018). AKRON: an Algorithm for Approximating Sparse Kernel Reconstruction. Signal Processing.

[12] Bayar B, Bouaynaya N, Shterenberg R. Kernel Reconstruction: an Exact Greedy Algorithm for Compressive Sensing. In: International Workshop on Genomic Signal Processing and Statistics: 2014.

[13] Khan J, Bouaynaya N, Fathallah-Shaykh H. Tracking of time-varying genomic regulatory networks with a lasso-kalman smoother. EURASIP J Bioinforma Syst Biol. 2014; 1. https://link.springer.com/article/10.1186/1687-4153-2014-3. 10.1186/1687-4153-2014-3 PMC3974129 24517200

[14] Fathallah-Shaykh HM, Bona JL, Kadener S (2009). Mathematical model of the drosophila circadian clock: Loop regulation and transcriptional integration. Biophys J.

[15] Xiong J, Zhou T. A kalman-filter based approach to identification of time-varying gene regulatory networks. PLoS ONE. 2013; 8(10). https://www.ncbi.nlm.nih.gov/pubmed/24116005. 10.1371/journal.pone.0074571 PMC3792119 24116005

[16] Rauch HE, Tung F, Striebel CT (1965). Maximum likelihood estimates of linear dynamic systems. AIAA J.

[17] Tibshirani R (1996). Regression shrinkage and selection via the lasso. J R Stat Soc.

[18] Arbeitman M, Furlong E, Imam F, Johnson E, Null B, Baker B, Krasnow M, Scott M, Davis R, White K (2002). Gene expression during the life cycle of drosophila melanogaster. Science.

[19] Marygold S, Leyland PC, Seal RL, Goodman JL, Thurmond J, Strelets VB, Wilson RJ (2013). Flybase: improvements to the bibliography. Nucleic Acids Res.

[20] Zhao W, Serpedin E, Dougherty E (2006). Inferring gene regulatory networks form time series data using minimum description length principle. Bioinformatics.

